# Determinants of prostate-specific antigen levels in African men

**DOI:** 10.1093/jncics/pkag087

**Published:** 2026-11-06

**Authors:** Eunsoo Cho, Ilir Agalliu, Thomas Rohan, Alfred I Neugut, Shakuntala Baichoo, Ianne Holanda Lauder, Akkshaya Rajesh, Hafees Ajibola, Oseremen Aisuodionoe-Shadrach, Pedro W Fernandez, Abraham C van Wyk, James E Mensah, Andrew Anthony Adjei, Afua O D Abrahams, Marjorie Ntiwa Quarchie, Akindele Olupelumi Adebiyi, Sikiru Adekola Adebayo, Olufemi Ogunbiyi, Mohamed Jalloh, Nana Yaa Snyper, Ben Adusei, Joseph Lachance, Timothy R Rebbeck

**Affiliations:** Department of Epidemiology, Harvard T.H. Chan School of Public Health, Boston, MA, United States; Department of Epidemiology and Population Health, Albert Einstein College of Medicine, Bronx, NY, United States; Department of Epidemiology and Population Health, Albert Einstein College of Medicine, Bronx, NY, United States; Department of Medicine, Vagelos College of Physicians & Surgeons, Columbia University, New York, NY, United States; Department of Epidemiology, Mailman School of Public Health, Columbia University, New York, NY, United States; Department of Digital Technologies, FoICDT, University of Mauritius, Reduit, Mauritius; Department of Biology, Georgia Institute of Technology, Atlanta, GA, United States; Department of Biology, Georgia Institute of Technology, Atlanta, GA, United States; Division of Urology, Stellenbosch University, Cape Town, South Africa; Division of Anatomical Pathology, National Health Laboratory Service, Faculty of Medicine and Health Sciences, Tygerberg Hospital, Stellenbosch University, Cape Town, South Africa; University of Abuja, University of Abuja Teaching Hospital & Cancer Science Centre, Abuja, Nigeria; Division of Urology, Stellenbosch University, Cape Town, South Africa; Division of Anatomical Pathology, National Health Laboratory Service, Faculty of Medicine and Health Sciences, Tygerberg Hospital, Stellenbosch University, Cape Town, South Africa; Department of Surgery, College of Health Sciences, University of Ghana Medical School, Korle Bu, Accra, Ghana; Department of Pathology, College of Health Sciences, University of Ghana Medical School, Korle Bu, Accra, Ghana; Department of Pathology, College of Health Sciences, University of Ghana Medical School, Korle Bu, Accra, Ghana; Department of Medical Laboratory Sciences, College of Health Sciences, Korle Bu, Accra, Ghana; College of Medicine, University of Ibadan and University College Hospital, Ibadan, Nigeria; College of Medicine, University of Ibadan and University College Hospital, Ibadan, Nigeria; College of Medicine, University of Ibadan and University College Hospital, Ibadan, Nigeria; Hôpital General Idrissa Pouye, Dakar, Senegal; 37 Military Hospital, Accra, Ghana; Department of Epidemiology, Harvard T.H. Chan School of Public Health, Boston, MA, United States; Department of Biology, Georgia Institute of Technology, Atlanta, GA, United States; Department of Biology, Georgia Institute of Technology, Atlanta, GA, United States; Department of Epidemiology, Harvard T.H. Chan School of Public Health, Boston, MA, United States; Department of Medical Oncology, Dana-Farber Cancer Institute, Boston, MA, United States

## Abstract

**Background:**

Prostate-specific antigen (PSA) is widely used for screening and diagnosis of prostate cancer. Variation in PSA levels is influenced by age, genetics, and other factors, yet this variation has not been well characterized in African men.

**Methods:**

We used data on serum PSA extracted from medical records from prostate cancer cases and controls collected as part of the Men of African Descent and Carcinoma of the Prostate (MADCaP) network. We evaluate the association of PSA levels with country of origin, age, body mass index (BMI), and polygenic risk score.

**Results:**

The median PSA levels were 41.8 (0.2-9696) ng/mL in 1907 prostate cancer cases and 1.235 (0.01-9.72) ng/mL in 902 controls. Higher PSA levels were associated with increasing age, lower BMI, higher Gleason score, and more advanced tumor stage in cases, explaining approximately 35% of variation in log-transformed PSA levels. In controls, 4.8% of PSA variation was explained by age.

**Conclusion:**

Our data in African men are consistent with non-African studies that report PSA levels increase with age, inversely with BMI (suggesting a PSA dilution effect or reverse causation), and with more aggressive disease in cases. These data may be used to help understand PSA distributions and in the future may guide screening practices in African men.

## Background

Prostate-specific antigen (PSA) is widely used for screening and diagnosis of prostate cancer. Variation in PSA levels is influenced by age, genetics, and other factors, yet this variation has not been well characterized in African men. Although there is no universally agreed-upon threshold for abnormal PSA levels because of the wide variation in levels, a common screening criterion that triggers additional follow-up is a PSA level >4 ng/mL^1^^,^^2^ Lower thresholds increase the likelihood of false-positive results.^3^ PSA thresholds should also be interpreted in the context of age, with different thresholds recommended for different age groups. For example, Matti et al.^4^ estimated age-specific references to be 2.1, 3.2, 4.9, and 6.5 ng/mL for men in their 40s, 50s, 60s, and 70s, respectively. Elevated PSA levels can vary by factors such as age, family history of prostate cancer, African ancestry, benign prostatic hypertrophy (BPH), prostatitis, and obesity.^5^ For example, in the United States, African American men and Caucasian men aged 60 with no family history of prostate cancer but the same PSA level of 3.5 ng/mL are expected to have different positive biopsy risk levels: 32% for Caucasian men and 39% for African American men.^6^

Despite the importance of PSA levels in prostate cancer diagnosis and risk assessment, there is limited knowledge about the age-specific variation of PSA levels, as well as the factors that may explain this variation, in African men.^7^ Both United States Preventive Services Task Force (USPSTF) and American Society of Clinical Oncology (ASCO) consider African descent to be a factor pointing toward starting prostate cancer screening at an earlier age.^8^^,^^9^ The categorization of African American men as a higher-risk population for prostate cancer is justified by higher rates of aggressive prostate cancer, suggesting PSA-based screening is potentially more beneficial to this cohort than to the general population. These epidemiological patterns reinforce the importance of studying PSA levels in men of African descent. To address this gap, this study aims to describe the age-specific distribution of PSA levels in prostate cancer cases and controls and identify factors that may explain PSA variation. Moreover, leveraging data from a case-control study in sub-Saharan Africa (SSA), multivariable linear regression models were fitted to measure the effect size of polygenic risk score (PRS), age, body mass index (BMI), and pathology on PSA level.

## Methods

### Study design

To examine variation in PSA, primary data were obtained from the Men of African Descent and Carcinoma of the Prostate (MADCaP) study. MADCaP is a multicenter Pan-African cancer research network comprising 7 centers in SSA. Study methods have been described previously.^10^ The SSA centers contributing to this study were University College Hospital (Ibadan, Nigeria), Ghana Prostate Cancer Study (Accra, Ghana), Korle-Bu Teaching Hospital (Accra, Ghana), 37 Military Hospital (Accra, Ghana), Institut de Formation et de la Recherche en Urologie (Dakar, Senegal), Tygerberg Hospital (Cape Town, South Africa), and University of the Witwatersrand (Johannesburg, South Africa).

MADCaP employs a case-control study design. Cases consist of patients newly diagnosed (within the past 12 months) between 2016 and 2020 through pathological examination and medical record review, excluding those previously diagnosed with prostate cancer. At least 1 control was frequency-matched to each case based on 5-year age groups, and these are recruited from nonurology and nononcology clinics at the same centers from which cases were identified. However, many controls did not have PSA measurements, and as a result the number of controls per case was limited.

PSA, PRS, age, BMI, and pathology data were used to examine the variation of PSA using ordinary least squares linear multiple regression models. While testing regression diagnostics, raw PSA reported extreme skewness, which violated linearity and normality assumptions for regression analyses (Figure S1). Therefore, PSA data were log-transformed before main analyses (Figure S1). Collinearity and homoscedasticity were ensured through residual plots, variance influence factor, and Breusch-Pagan test. Residuals from regression models were also plotted to ensure normality and linearity of regression models fitting log-transformed PSA (Figure S2).

Age was recorded at the time of diagnosis and coded into 5-year categories from 30-34 years to 95-99 years. Controls’ ages were frequency-matched to cases from the SSA clinics. BMI served as an obesity measure and was calculated from individual-level height and weight data using the formula: $\mathrm{BMI}=\frac{\mathrm{weight}\;(kg)*10,000\;}{\mathrm{height}\;{\left(cm\right)}^{2}}$. PSA levels were specified in numbers with ng/mL units. Three pathology variables were also included: Grade Group with values of 1-5; BPH where 0 represents No and 1 represents Yes; and the clinical summary stage of the primary tumor with stage T1-T4.

### Genotyping and imputation

Genotype data from a prior MADCaP Network study^10^ were available for 2505 prostate cancer cases and 2222 controls individuals from Senegal, Nigeria, Ghana, and South Africa. These participants were genotyped using the MADCaP Array, also known as the Axiom Pan-African Cancer Research Array.^11^ As described previously,^10^ markers with low call rates were excluded, as were markers that failed Hardy-Weinberg tests. Additional quality control filters included the exclusion of related individuals, individuals with XXY karyotype. All men were self-identified as native African, and men who reported their ancestors were non-African were excluded from the study. No analysis of African ancestry was undertaken. MADCaP Network genotype data used in this study are publicly available via dbGAP (accession: phs002718.v1.p1).

We performed genomic imputation to maximize the overlap between the MADCaP Network genetic dataset and known PSA associations. The TOPMed Imputation Server was used (https://imputation.biodatacatalyst.nhlbi.nih.gov/), as was the TOPMed-r3 reference panel.^12^^,^^13^ This imputation server and reference panel have been shown to be effective for African datasets.^14^ Imputation was conducted on all chromosomes using the full set of genotyped markers.

### Polygenic risk score

This analysis used a curated set of 128 PSA-associated loci from PGS Catalog entry PGS003378 (https://www.pgscatalog.org/score/PGS003378/). These PSA-associated variants were identified via a large multi-ancestry meta-analysis.^15^ After filtering the genotype file to exclude low imputation quality variants (R² ≤ 0.8), the final risk score calculation incorporated 103 variants. Individual risk scores were calculated using genotypes represented as allele dosages (0, 1, or 2), weighted by their corresponding effect sizes, employing the PLINK 1.9 toolset.^16^

### Statistical analyses

The goals of our analyses were 2-fold. First, we describe the age-specific distribution of PSA levels fitting box plots in case and control groups. Second, we evaluate the relationship of PSA levels and risk factors, by fitting ordinary least-squares multiple linear regression models. These variables included PSA PRS, age, and BMI, while controlling for pathology characteristics (in the case group). All analyses were conducted in R (version 4.3.2). The following models fit separately for cases and controls were:


$$*\;\mathrm{Model}\;1:\hat{\log\;\left(\mathrm{PSA}\right)}=\beta_{0}+\beta_{1}\times \mathrm{PRS}+\beta_{2}\times \mathrm{Age}\;\mathrm{Group}+\epsilon$$

$$*\;\mathrm{Model}\;2:\hat{\log\;\left(\mathrm{PSA}\right)}=\beta_{0}+\beta_{1}\times \mathrm{Age}\;\mathrm{Group}+\beta_{2}\times \mathrm{BMI}+\beta_{3}\times \mathrm{Gleason}+\beta_{4}\times \mathrm{Staging}+\beta_{5}\times \mathrm{BPH}+\epsilon$$

$$*\;\mathrm{Model}\;3:\hat{\log\left(\mathrm{PSA}\right)}=\beta_{0}+\beta_{1}\times \mathrm{PRS}+\beta_{2}\times \mathrm{BMI}+\beta_{3}\times \mathrm{Gleason}+\beta_{4}\times \mathrm{Staging}+\beta_{5}\times \mathrm{BPH}+\epsilon$$

$$*\;\mathrm{Model}\;4:\hat{\log\left(\mathrm{PSA}\right)\;}=\beta_{0}+\beta_{1}\times \mathrm{PRS}+\beta_{2}\times \mathrm{Age}\;\mathrm{Group}+\beta_{3}\times \mathrm{BMI}+\beta_{4}\times \mathrm{Gleason}+\beta_{5}\times \mathrm{Staging}+\beta_{6}\times \mathrm{BPH}+\epsilon$$

$$\mathrm{Model}\;5:\hat{\log\left(\mathrm{PSA}\right)}=\beta_{0}+\beta_{1}\times \mathrm{Age}\;\mathrm{Group}+\beta_{3}\times \mathrm{BMI}+\epsilon$$

$$\mathrm{Model}\;6:\hat{\log\left(\mathrm{PSA}\right)}=\beta_{0}+\beta_{1}\times \mathrm{PRS}+\beta_{2}\times \mathrm{BMI}+\epsilon$$

$$\mathrm{Model}\;7:\hat{\log\left(\mathrm{PSA}\right)}=\beta_{0}+\beta_{1}\times \mathrm{PRS}+\beta_{2}\times \mathrm{Age}\;\mathrm{Group}+\epsilon$$

$$\mathrm{Model}\;8:\hat{\log\left(\mathrm{PSA}\right)}=\beta_{0}+\beta_{1}\times \mathrm{PRS}+\beta_{2}\times \mathrm{Age}\;G\mathrm{roup}+\beta_{3}\times \mathrm{BMI}+\epsilon$$

*Model fitted for cases only.

Models 4 and 8 are full multivariate models for the case and control group. Models 1-3 and 5-7 are exploratory models testing the effect of independent variables on PSA level with different covariate combinations. Regression coefficients, standard errors, R-squared values, and *P*-values were computed.

## Results

Figure 1 presents the sample inclusions and exclusions applied to reach the final sample size of 2809. Missing data and outliers were identified and removed. PSA levels >10 ng/mL were excluded in controls (ie, assuming these men may have had undiagnosed prostate cancer); PSA values >10 000 ng/mL for cases were excluded as outliers. A 10 ng/mL threshold was considered instead of 4 ng/mL to minimize outliers being removed, given that many with higher PSA levels between 4 and 10 ng/mL may not have prostate cancer.^17^ A total of 92 controls had PSA levels higher than 4 ng/mL and lower than 10 ng/mL. Additionally, BMI >40 values were excluded as outliers.

**Figure 1.**
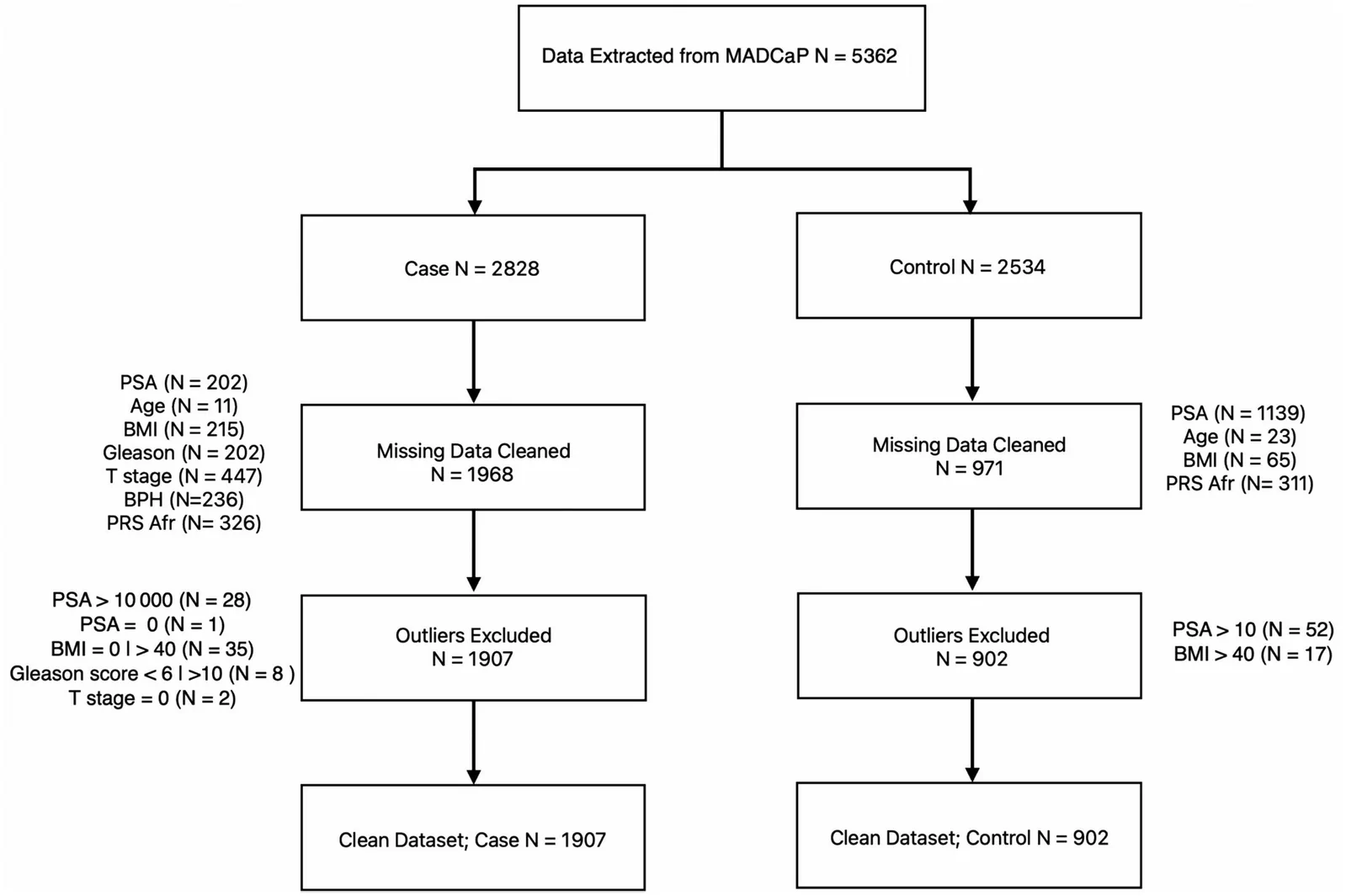
Flowchart of inclusion and exclusion criteria.

In total, 860 cases and 1563 controls with 1 or more missing data and additional outliers of 61 cases and 69 controls were removed. A total of 1139 out of 2534 controls were missing PSA level data. Upon these criteria identification, individuals with 1 or more missing data points or outliers were also excluded from analyses. The final, clean dataset contains 1907 cases and 902 controls.

Table 1 presents descriptive statistics separately for cancer cases and controls. The median PSA levels are significantly higher in cases (41.83) compared with controls (1.24). The log-transformed PSA values also reflect this difference, with cases having a median of 3.73 vs 0.21 in controls. PSA PRS and BMI values show less pronounced differences between cases and controls, with similar central tendency measures in both groups.

**Table 1.** Descriptive statistics.

	Case (*N* = 1907)						Control (*N* = 902)					
	Min.	Q1	Median	Mean	Q3	Max.	Min.	Q1	Median	Mean	Q3	Max.
**PSA**	0.20	15.38	41.83	417.21	185.10	9696	0.010	0.700	1.235	1.818	2.290	9.720
**Log PSA**	−1.609	2.733	3.734	4.146	5.221	9.179	−4.605	−0.367	0.211	0.292	0.826	2.274
**PRS**	3.165	3.704	3.874	3.882	4.052	4.650	3.101	3.710	3.885	3.878	4.045	4.57
**BMI**	13.5	21.88	24.84	25.27	28.27	39.78	14.20	22.15	25.10	25.49	28.58	39.45

	Freq	%	Freq	%
**Age group**				
30-39	2	0.10	14	1.55
40-49	25	1.31	53	5.88
50-59	273	14.31	221	24.50
60-69	805	42.21	412	45.68
70-79	684	35.86	163	18.07
80-89	113	5.92	37	4.10
90+	5	0.26	2	0.22
**Grade Group**				
1	337	17.67		
2	469	24.59		
3	364	19.08		
4	298	15.62		
5	439	23.02		
**T Stage**				
1	690	36.18		
2	812	42.57		
3	282	14.79		
4	123	6.45		
**Benign Hypertrophic Hyperplasia (BPH)**				
0	1574	82.54		
1	284	14.89		
2	49	2.57		

Table 1 provides descriptive statistics of raw and log-transformed PSA levels and the covariates used in the analysis. PSA levels exhibited a wide, skewed variation, with a median of 41.83 and a mean of 417.21 in the case group. Median PSA level for the control group was 1.24, with a mean of 1.82. This skewness is adjusted upon log-transformation of PSA level. Histograms and quantile plots in Figure S2 of the case/control regression model validates normality and linearity of the models.

### Explaining the distribution of PSA levels

Figure 2 presents the results from the age-specific distribution of PSA levels in prostate cancer cases and controls. The box plots demonstrate that PSA levels increase with age in both cases and controls. This upward trend was similar between raw and log-transformed PSA, although untransformed PSA levels represented a very skewed distribution. PSA levels were higher in the case group than in the control group in all age groups. In addition, the increase in PSA in the oldest age groups was not apparent: 85+ for cases and 90+ for controls. This slight decline might be due to healthy survivor effect.^18^ This finding aligns with trends observed in other literature, indicating that PSA levels tend to rise with age.

**Figure 2.**
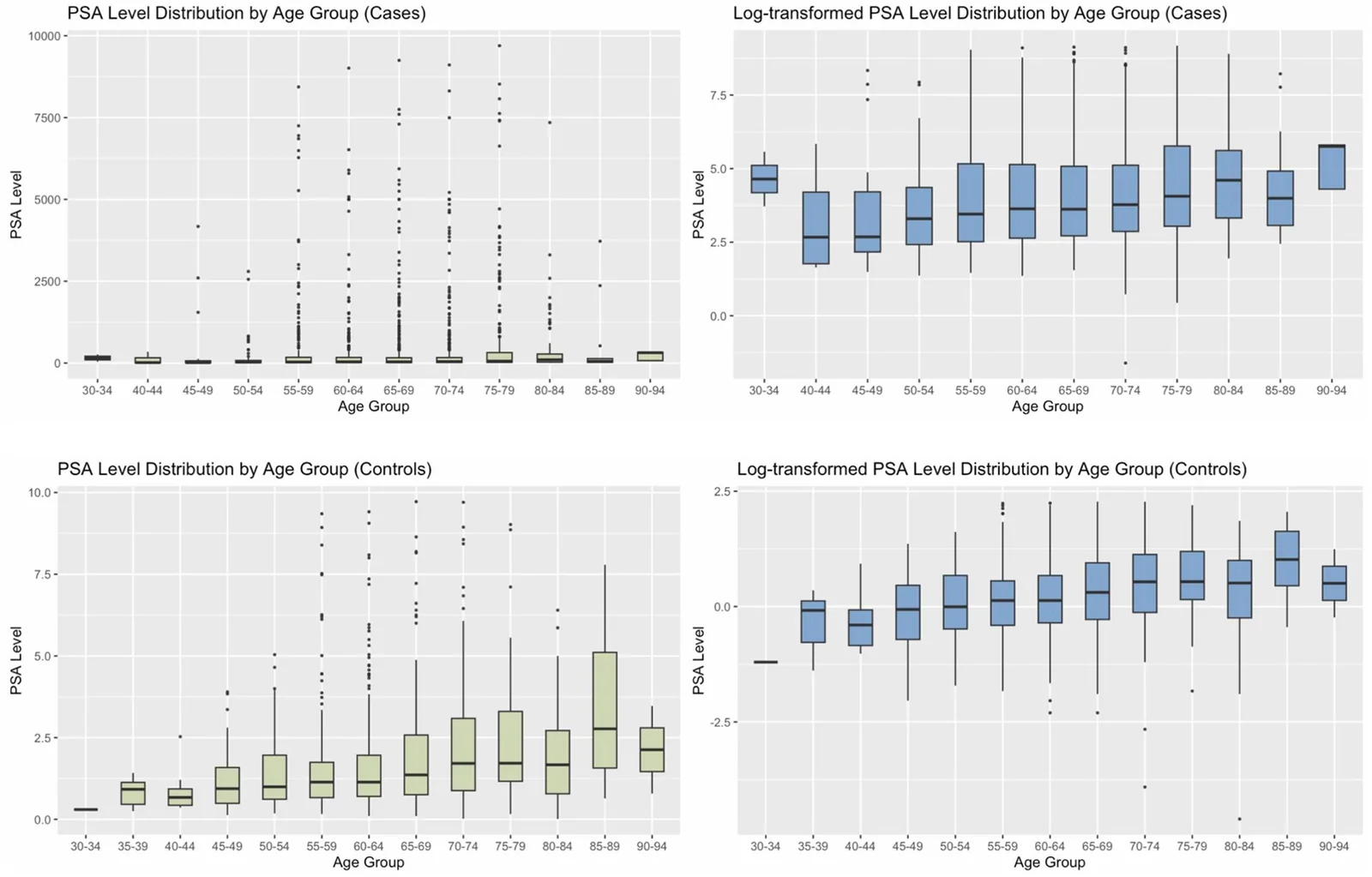
Box plots of age-specific PSA levels by case and control group (*N* = 2809). **A**) Raw PSA values in cases. **B**) Raw PSA values in controls. **C**) Log-transformed PSA values in cases. **D**) Log transformed PSA values in controls.

From the regression models (Table 2), age group, Gleason score grade group, and staging are positively associated with log-transformed PSA level among cases on a multiplicative scale. BMI and BPH are negatively associated with log-transformed PSA level among cases on a multiplicative scale. Age is also associated with PSA levels on a multiplicative scale among controls. The R-squared value of 0.346 from the case group indicates that a substantial proportion of variance in log-transformed PSA levels was explained by the predictors included in the model. However, the R-squared value from the control group was 0.048, suggesting other factors are associated with PSA variation in African men without prostate cancer.

**Table 2.** Regression coefficient (β) and SE estimates from linear regression models for log-transformed PSA levels on age group, polygenic risk score (PRS), body mass index (BMI), Grade Group, stage, and BPH in cases and controls.

	Cases (*N* = 1907)				Controls (*N* = 902)			
	(1)	(2)	(3)	(4)	(5)	(6)	(7)	(8)
**Age Group**	0.122*** (0.025)	0.073*** (0.020)		0.073*** (0.020)	0.102*** (0.015)		0.101*** (0.015)	0.102*** (0.015)
**PRS**	−0.294 (0.164)		−0.143 (0.134)	−0.140 (0.134)		0.194 (0.117)	0.210 (0.115)	0.208 (0.115)
**BMI**		−0.053*** (0.007)	−0.055*** (0.007)	−0.053*** (0.007)	0.007 (0.006)	0.006 (0.006)		0.007 (0.006)
**Grade Group**		0.393*** (0.025)	0.396*** (0.025)	0.392*** (0.025)				
**Stage**		0.731*** (0.004)	0.733*** (0.040)	0.730*** (0.040)				
**BPH**		−0.289*** (0.073)	−0.293*** (0.073)	−0.291*** (0.073)				
**Intercept**	4.292*** (0.671)	2.355*** (0.283)	3.545*** (0.575)	2.910*** (0.601)	−0.688*** (0.197)	−0.677 (0.482)	−1.319** (0.462)	−1.493*** (0.487)**
**R^2^**	0.013	0.346	0.342	0.346	0.045	0.002	0.047	0.048

* *P* < .05.

** *P* < .01.

*** *P* < .001.

PSA levels increased 1.08-fold for each 1-unit increase in age group among cases, after controlling for PSA, PRS, BMI, and pathology variables (*t* = 3.553, *P* < .001). For each 1-unit increase in BMI, the PSA level is 0.95 times lower, holding other covariates constant (*t* = -7.293, *P* < .001). With each 1-unit increase in the Gleason grade group, the PSA level is 1.48 times higher, holding other covariates constant (*t* = 16.004, *P* < .001). For each 1-unit increase in T stage, the PSA level is 2.07 times higher, holding other covariates constant (*t* = 18.041, *P* < .001). Having BPH is also associated with 0.75 times lower levels of PSA, holding other covariates constant (*t* = -3.984, *P* < .001). There is not sufficient evidence to suggest an association between PRS and the PSA levels (*t* = -1.046, *P* = .296).

For controls, with a 1-unit increase in age group, the PSA level is 1.107 times higher among cases after controlling for BMI and PSA PRS (*t* = 6.646, *P* < .001). Although not statistically significant at the conventional alpha level of 0.05, there is a suggestive trend where a 1-unit increase in PSA PRS is associated with a 1.231 times higher PSA level after holding other covariates constant (*t* = 1.810, *P* < .1). There is no statistically significant association between BMI and the PSA levels in our sample (*t* = 1.140, *P* = .254). These regression results are consistent before and after removing missing data and outliers (results not shown).

We observed a modest inverse relationship between PSA and PRS in cases (nonparametric correlation *r* = -0.031; Figure S3a) and a modest direct association of PSA and PRS in controls (*r* = 0.023; Figure S3a). We did not observe a consistent relationship between PRS and PSA by age group (Figure S3c-d).

## Discussion

This article uses a Pan-African case-control study to (a) explain the distribution of PSA levels among men of African descent and (b) test the risk factors of prostate cancer for elevated PSA levels in the sub-Saharan African context using multivariable linear regression models. In the case group, we found that consensus risk factors for prostate cancer, including age, obesity, and pathology, were all associated with higher PSA levels. Although in the control group, the proportion of variation the model explained was 4.8%, age was found to have a statistically significant association with PSA levels.

This study adds empirical evidence to consensus knowledge about prostate cancer and PSA among African men. Figure 2 displays a consistent upward pattern until the oldest age groups. This is a consensus pattern tendency worldwide.^19-22^ Furthermore, higher PSA levels in the case group than in the control group supports findings from most studies, including in US Black men.^23^ In addition to identifying age-group-specific PSA levels in SSA contexts, the cross-national multivariate regression analysis revealed how age, BMI, and pathology are associated with elevated PSA levels. In addition, BMI from the control group did not have a statistically significant association with elevated PSA level after controlling for age and PRS. Although not applicable to both groups, an inverse association between BMI and PSA level in the case group was observed, as seen in other populations.^24^^,^^25^ This observation may be explained either by PSA dilution among men with higher BMI or reverse causation if men in ill health lose weight yet have very aggressive disease associated with higher PSA levels. The inconsistency in the control group may be due to low R-squared.

This article also has limitations that should be addressed in the future. First, participants with missing PSA data were removed from analysis. With 64% of the control group being removed because of missing PSA-level data, this may induce bias in which controls had PSA data. Twenty-eight controls with >10 ng/mL PSA may have also been misclassified (eg, have not undergone diagnostic workup or were misdiagnosed with other prostatic pathologies). Second, MADCaP is a hospital-based case-control study with its embedded limitation. Population-based studies may be able to provide additional insights into the relationship between elevated PSA levels and risk factors in men of African descent. Third, the PSA PRS was not associated with PSA in our study. An explanation for this observation is that the previously constructed PSA PRS is not portable to African populations. Most PSA PRS were developed using samples in which PSA screening was prevalent (eg, in the United States).^15^ Among cases, PSA levels are influenced by disease characteristics and progression at diagnosis, which may outweigh the relatively modest contribution of germline PSA-associated variants. Fourth, we recorded the use of BPH medications including finasteride and dutasteride. Only 9 (0.17%) men had finasteride and none had dutasteride exposures out of 5368 cases and controls that had a clinician note or medical records abstraction. Thus, we did not correct for this exposure in the analysis because of the very low usage of these agents in our African dataset. Finally, we evaluated whether there was evidence for reverse causation of BMI and PSA because low BMI and elevated PSA may reflect consequences of occult advanced prostate cancer rather than antecedent risk factors. To assess this possibility, we abstracted evidence of metastatic disease at diagnosis using TNM/AJCC stage and treatment records. We performed sensitivity analyses excluding cases with stage IV disease and restricting analyses to localized prostate cancer. Attenuation of associations after these exclusions could be interpreted as evidence consistent with reverse causation. The r^2^ for the association after removing stage IV cases dropped only slightly from 0.346 to 0.289, and the *P*-value association remained statistically significant. Thus, the potential for reverse causation in our analysis is low, and it does not negate the association we report here.

Despite these limitations, the results provide information that may inform clinical practice to optimize prostate cancer screening and potentially minimize disparities; they may inform policy decisions regarding screening. Age- and risk group-specific distribution of PSA as a risk factor can provide evidence in crafting guidelines. Understanding the variation of PSA and how it interplays with risk factors can create a system to weigh data points accordingly to optimize personalized or group-specific recommendations.^26^ Refining cut-points for prostate cancer detection strategies can limit overdiagnosis in prostate cancer if screening is implemented.^27^

## Supplementary Material

pkag087_Supplementary_Data

## Data Availability

The genomic data underlying the PRS used in this article are available through dbGaP Study Accession: phs002718.v1.p1 (https://www.ncbi.nlm.nih.gov/projects/gap/cgi-bin/study.cgi?study_id=phs002718.v1.p1). Other data are available upon request from the corresponding author using a controlled access mechanism as required by human subjects study approvals.
